# Influence of exogenous and endogenous estrogen on thermoregulatory responses to mild heat and the interaction with light and dark phases

**DOI:** 10.1186/s12576-020-00782-x

**Published:** 2020-11-30

**Authors:** Shuri Marui, Yuta Masuda, Issei Kato, Kei Nagashima

**Affiliations:** 1 Body Temperature and Fluid Laboratory, Faculty of Human Sciences, Waseda University, Mikajima 2-579-15, Tokorozawa, Saitama 359-1192 Japan; 2 Graduate School of Human Sciences, Faculty of Human Sciences, Waseda University, Tokorozawa, Japan

**Keywords:** Female hormones, Circadian rhythm, Autonomic thermoregulation, Core temperature, Hot environment

## Abstract

The present study aimed to determine the influence of estradiol (E_2_) and the interaction with circadian phases on thermoregulatory responses to mild heat in female rats. Heat loss and production during 3-h exposure to the environment at an ambient temperature of 28–34 °C were assessed by measuring abdominal temperature (*T*_abd_), tail skin temperature, and oxygen consumption in ovariectomized rats with and without E_2_ replacement (OVX + E_2_ and OVX, respectively) and in control rats in the proestrus (P) and diestrus (D) phases. In the light phase, *T*_abd_ remained unchanged in all groups. *T*_abd_ increased in the dark phase, but was lower in the OVX + E_2_ and P groups than in the OVX and D groups. Oxygen consumption decreased at 34 °C, but to a lesser extent in the OVX + E_2_ group than in the OVX group. These results suggest that E_2_ activates thermoregulation in mild heat in the dark phase.

## Background

Several studies have reported that estradiol (E_2_) activates thermoregulatory mechanisms during exposure to heat in female rats [1–5]. Baker et al. [1] demonstrated that, in the extreme heat of 38 °C, the increase in body temperature was greater in ovariectomized (OVX) rats than in OVX rats with E_2_ replacement (OVX + E_2_). They suggested that reduced evaporative heat loss in OVX rats is involved in the mechanism. However, Dacks and Rance [2] reported that the increase in body temperature was greater in OVX rats than in OVX + E_2_ even at an ambient temperature (*T*_a_) of 32.5 °C, a temperature at which evaporative heat loss is less involved in thermoregulation [6]. They also reported that dry heat loss from the tail was greater in OVX rats than in OVX + E_2_ rats. Hosono et al. [3] reported that, at *T*_a_ of 32–36 °C, heat-escape behavior did not differ between OVX and OVX + E_2_ rats. These results suggest that E_2_ activates neither the heat loss response of the tail nor thermoregulatory behavior in mild heat. Thus, it remains unclear how E_2_ attenuates the increase in body temperature in mild heat. Previous studies have reported that acute and chronic heat exposure suppress metabolism with attenuation of thyroid function [7, 8] and/or decreased spontaneous activity [9]. The results suggest that the attenuation of metabolism is part of the thermoregulatory response to heat, although it is unclear whether E_2_ is involved in the underlying mechanism.

Endothermic animals exhibit circadian changes in body temperature, metabolism, and spontaneous activity. In addition, some studies have demonstrated the involvement of E_2_ in these changes. For example, body temperature is lower in OVX rats than in OVX + E_2_ rats in the middle of the dark phase [10]. Further, Williams et al. [11] reported that E_2_ reduces the tail skin temperature in the dark phase, indicating the heat loss response of the tail. These results suggest that, when evaluating the influence of E_2_ on thermoregulatory responses to heat, we need to consider the concurrent influence of circadian changes. However, no studies have yet evaluated this influence. Therefore, the aim of this study was to identify the mechanism by which E_2_ activates thermoregulatory responses during exposure to mild heat in female rats, and to determine whether the influence of E_2_ differs between circadian cycles. Therefore, we exposed OVX rats with and without E_2_ replacement to the environment at 28–34 °C and compared the body temperature and heat loss and metabolic responses between the light and dark phases. Moreover, to know if physiological change in plasma E_2_, normally observed within the estrus cycle, also affect the responses to the mild heat, the same heat exposure was conducted in the control female rats with the proestrus and diestrus phases, in which plasma E_2_ is higher and lower, respectively.

## Methods

### Animals

Adult virgin female Wistar rats (*n* = 48; body weight, 249 ± 25 g [mean ± standard deviation]); age, 9–11 weeks; Takasugi Experimental Animals Supply, Saitama, Japan) were used in the present study. They were housed individually in plastic cages (45 cm × 25 cm × 20 cm) at a *T*_a_ of 25 °C under a 12/12 h light/dark cycle (lights on at 07:00). Food and water were freely available. Animal experiments and care were conducted in accordance with the institutional guidelines, which follow the Fundamental Guidelines for Proper Conduct of Animal Experiments and Related Activities in Academic Research Institutions under the jurisdiction of the Ministry of Education, Culture, Sports, Science, and Technology (Notice No. 71, 2006; Tokyo, Japan). The Institutional Animal Care and Use Committee of Waseda University (Tokyo, Japan) approved all experimental procedures applied in the present study (Approval No. A071).

### Surgery

Rats were divided to two groups that were used in two different experiments (i.e., *Experiments* *1* and *2*; *n* = 28 and 20, respectively). Under inhalation anesthesia with 2% isoflurane (Abbott Japan, Tokyo, Japan) in air, a radio-transmitter device with two wire-type thermistors was placed in the abdominal cavity of each rat to measure abdominal temperature (*T*_abd_), tail temperature (*T*_tail_), and spontaneous activity (3.5 cm^3^, 7.5 g; F40-TT transmitter; Data Sciences International, New Brighton, MN, USA) as previously described [12]. Briefly, having been passed through the muscle layer and subcutaneous tissue of the abdomen, the tip of one wire was placed under the skin of the lateral tail, 2 cm beyond the tail base. The other wire was fixed in the abdominal cavity. Spontaneous activity was estimated by the relative change in signal strength from the transmitter.

In *Experiment* *1*, the rats were bilaterally ovariectomized with a retroperitoneal approach. A silicone tube (inner diameter, 1.57 mm; outer diameter, 3.18 mm; length, 30 mm; Kaneka, Osaka, Japan) was placed in the subcutaneous tissue of the right side of the back, which was filled with E_2_ powder (50–60 mg; Sigma-Aldrich, St. Louis, MO, USA; OVX + E_2_, *n* = 14) or not filled with E_2_ powder (OVX, *n* = 14). E_2_ is permeable to silicone and the placement provided a constant level of plasma E_2_ for > 14 days in OVX rats [13, 14]. The rats recovered from the surgery after ≥ 14 days. Penicillin G (1000 U; Meiji Pharmaceutical, Tokyo, Japan) was subcutaneously injected to prevent postsurgical infection.

In *Experiment* *2*, a sham operation of the bilateral ovariectomy was performed. During the recovery period, vaginal smears from the rats were obtained every morning for ≥ 10 days, and the estrus cycle was determined [15]. Rats exhibiting a regular estrus cycle of 4–5 days were used for further experiments.

### Exposure to the environment at 28 °C, 31 °C, and 34 °C

*T*_abd_, *T*_tail_, and spontaneous activity were recorded every 60 s with a data collection system (Dataquest ART; Data Sciences International). We verified that each rat showed clear circadian changes of these parameters. Then, each rat was moved to a Plexiglas box (35 cm × 20 cm × 20 cm) in a climatic chamber (Program Incubator IN604; Yamato Scientific, Tokyo, Japan), where oxygen consumption ($\dot{\text {V}}$O_2_) was determined by indirect calorimetry. The box was attached to an airflow system with a flow rate of 2.0 l min^−1^. The difference in oxygen tension between room air and the air that passed through the chamber was determined every 60 s with an electrochemical oxygen analyzer (model LC-700E; Toray, Tokyo, Japan). $\dot{\text {V}}$O_2_ was calculated as the product of the difference in oxygen tension and the airflow rate. The value was divided by 0.75 power of the body weight (i.e., Brody–Kleiber formula [16]) and corrected to the standard temperature and pressure dry condition. The chamber was maintained at 25.0 ± 0.2 °C and the *T*_a_ was continuously recorded. For 3 days, the rats were housed in this condition, and the data on the last day were used as the control. In *Experiment* *1*, at 9:30 or 21:30 on the 4th day (exposure day), the rats were exposed to the environment at 28 °C, 31 °C, and 34 °C for 1 h in sequence. The period in each phase was selected because *T*_abd_, *T*_tail_, and spontaneous activity are less influenced by the circadian fluctuations based on our preliminary finding. In *Experiment* *2*, the day of the exposure was selected in the proestrus phase (P group, *n* = 10) or the diestrus phase (D group, *n* = 10). In both experiments, food and water were removed 2 h before heat exposure. Body weight was measured before and after exposure.

### Blood analysis

After completion of the final heat exposure period, the rats were euthanized with an intraperitoneal injection of overdose pentobarbital sodium (100 mg kg^−1^ body weight: Kyoritsu Seiyaku, Tokyo, Japan). A 2 ml blood sample was obtained from the right ventricle and centrifuged at 4 °C, and the plasma was stored at − 80 °C until use. The estradiol level in the plasma was determined using an enzyme-linked immunosorbent assay kit (Estradiol EIA Kit; Cayman Chemical, Ann Arbor, MI, USA). The detection limit of estradiol was 20 pg ml^−1^. The coefficient of variation of the measurement was < 13%.

### Calculation and statistics

The sample size was determined using G*Power 3.1.9.2 (Heinrich-Heine-University of Düsseldorf, Düsseldorf, Germany) [17]. To evaluate the parameters during heat exposure, we used an effect size of 0.4, an *α* error probability of 0.05, and a power (1 − *β*) of 0.8. We estimated that the required sample size was at least five rats in each group.

The values for *T*_abd_, *T*_tail_, spontaneous activity, and $\dot{\text {V}}$O_2_ during heat exposure were averaged every 30 min. $\dot{\text {V}}$O_2_ was corrected by body weight, which was averaged by the initial and final body weight. Thermal conductance from the body core to the environment (thermal conductance of the whole body) was calculated as $\dot{\text {V}}$O_2_/(*T*_abd_ − *T*_a_) [18, 19]. The heat loss index of the tail was estimated as (*T*_tail_ − *T*_a_)/(*T*_abd_ − *T*_a_) [20].

A two-way ANOVA or two-way ANOVA with repeated measurement (group × time) was performed to compare the values of the heat exposure day among the groups. When a significant difference was observed, post hoc Bonferroni tests were conducted. The null hypothesis was rejected at *P* < 0.05. IBM SPSS Statistics for Windows (version 25.0.; IBM Corp., Armonk, NY, USA) was used for statistical analysis. All values are presented as means ± standard error.

## Results

### Body weight and plasma E_2_ level

Table 1 presents the initial and final body weight and plasma E_2_ level. In *Experiment* *1*, there was a significant effect of time [*P* < 0.001, *F*_(1, 24)_ = 19.22] in body weight. In addition, a significant interaction between time and group was observed [*P* < 0.001, *F*_(3, 24)_ = 8.76]. The final body weight was greater than the initial body weight in both phases in the OVX group (*P* < 0.001). Because of difference in recovery period from the surgery and adjustment of the estrus phase, the initial body weight on the exposure day varied in each group.

**Table 1:** Body weight and plasma E_2_ level in *Experiments* *1* and *2*

	Group	Initial body weight, g		Final body weight, g		Plasma E_2_ level, pg ml^−1^	
		Light phase	Dark phase	Light phase	Dark phase	Light phase	Dark phase
*Experiment* *1*	OVX	240 ± 20	254 ± 23	262 ± 17^‡^	273 ± 21^‡^	47 ± 6	53 ± 4
	OVX + E_2_	228 ± 9	252 ± 32	244 ± 13	258 ± 21	142 ± 25*	159 ± 31*
*Experiment* *2*	D	234 ± 40	224 ± 7	242 ± 26	240 ± 6	47 ± 6	43 ± 12
	P	250 ± 24	256 ± 26	254 ± 10	261 ± 16	125 ± 29^†^	119 ± 6^†^

Data are presented as means ± standard error

OVX, ovariectomized; E_2_, estradiol; D, control rats in the diestrus phase; P, control rats in the proestrus phase

* Significant difference between the OVX and OVX + E_2_ groups (*P* < 0.05)

^†^ Significant difference between the D and P groups (*P* < 0.05)

^‡^ Significant difference from the initial body weight (*P* < 0.05)

A significant effect of group [*P* < 0.001, *F*_(3, 20)_ = 14.35] was observed in plasma E_2_. In *Experiment* *1*, the plasma E_2_ level was lower in the OVX group than the OVX + E_2_ group in both phases. In *Experiment* *2*, the plasma E_2_ level was lower in the D group than the P group in the two phases. There were no differences between the OVX and D groups and the OVX + E_2_ and P groups in each phase.

### *T*_abd_, *T*_tail_, spontaneous activity, and $\dot{\text {V}}$O_2_ on the control day

**Fig. 1:**
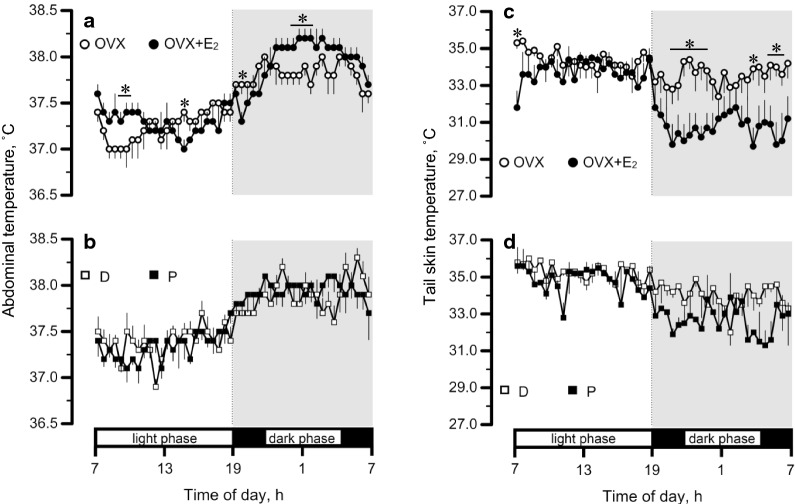
Circadian changes of abdominal temperature (*T*_abd_) and tail temperature (*T*_tail_) in the ovariectomized (OVX) and OVX + estradiol (E_2_) groups (open and closed circles, respectively), and in the D and P groups (open and closed squares, respectively). The data were obtained while rats were housed in the home cages. Data are presented as means ± standard error (**a** and **c**, *n* = 7 in each group; **b** and **d**, *n* = 5 in each group). *Significant difference between the OVX and OVX + E_2_ groups (*P* < 0.05). D, control rats in the diestrus phase; P, control rats in the proestrus phase

Figure 1 illustrates the circadian changes of *T*_abd_ and *T*_tail_ before the 4-day protocol (i.e., those in home cages) in *Experiments* *1* and *2*, shown in 30-min bins. In *Experiment* *1*, there were significant interaction between time and group in *T*_abd_ [*P* < 0.001, *F*_(47, 564)_ = 2.8]. *T*_abd_ was lower in the OVX group than the OVX + E_2_ group at 9:00–9:30 and 0:00–1:30 (*P* < 0.05). There were significant interaction between time and group in *T*_tail_ [*P* < 0.001, *F*_(47, 564)_ = 3.2]. *T*_tail_ was higher in the OVX group than the OVX + E_2_ group at 20:30–23:30 and 5:00–6:00 (*P* < 0.05).

Table 2 summarizes the circadian changes of *T*_abd_, *T*_tail_, spontaneous activity, and $\dot{\text {V}}$O_2_ on the control day by calculating (i) the averages of the whole period and the light and dark phases, and (ii) the circadian amplitude (i.e., the difference between the maximum and minimum values). In *Experiment* *1*, there were significant effects of phase [*P* = 0.003, *F*_(1, 12)_ = 13.63] and group [*P* = 0.032, *F*_(1, 12)_ = 5.89] with a significant interaction between the two factors [*P* = 0.039, *F*_(1, 12)_ = 5.34] in *T*_tail_. *T*_tail_ was higher in the OVX group than the OVX + E_2_ group in the dark phase (*P* = 0.011). The amplitude was smaller in the OVX group than the OVX + E_2_ group (*P* = 0.006). In *Experiment* *2*, there were significant effects of phase [*P* < 0.001, *F*_(1, 8)_ = 62.51] and group [*P* = 0.008, *F*_(1, 8)_ = 12.36] with a significant interaction between these two factors [*P* = 0.04, *F*_(1, 8)_ = 6.03] in *T*_tail_. In the dark phase, *T*_tail_ was higher in the D group than the P group (*P* = 0.002). The amplitude was lower in the D group than the P group (*P* = 0.029). *T*_abd_, spontaneous activity, and $\dot{\text {V}}$O_2_ were higher in the dark phase than the light phase in all groups. In the OVX + E_2_ and P groups, *T*_tail_ was lower in the dark phase than the light phase.

**Table 2:** Average measurements of the whole day and the light and dark phases and the circadian amplitude on the control day

Group	Average of the whole day	Average of the light phase	Average of the dark phase	Amplitude
*T*_abd_, °C				
OVX	37.5 ± 0.1	37.3 ± 0.1	37.8 ± 0.1^§^	0.6 ± 0.1
OVX + E_2_	37.6 ± 0.1	37.3 ± 0.1	37.9 ± 0.1^§^	0.6 ± 0.1
D	37.8 ± 0.1	37.4 ± 0.1	37.9 ± 0.1^§^	0.5 ± 0.1
P	37.6 ± 0.1	37.3 ± 0.1	37.9 ± 0.1^§^	0.6 ± 0.1
*T*_tail_, °C				
OVX	34.0 ± 0.6	34.4 ± 0.4	33.5 ± 0.7	0.9 ± 0.3
OVX + E_2_	32.3 ± 0.7	33.8 ± 0.5	29.7 ± 0.8*^§^	3.4 ± 0.7*
D	34.6 ± 0.3	35.3 ± 0.2	34.0 ± 0.4	1.3 ± 0.2
P	33.7 ± 0.3	34.8 ± 0.3	32.7 ± 0.3^†§^	2.1 ± 0.1^†^
Spontaneous activity, au				
OVX	2.0 ± 0.2	1.1 ± 0.2	2.9 ± 0.4^§^	1.8 ± 0.4
OVX + E_2_	2.4 ± 0.4	1.4 ± 0.3	3.3 ± 0.5^§^	1.8 ± 0.3
D	1.5 ± 0.4	1.0 ± 0.4	2.7 ± 0.3^§^	1.8 ± 0.2
P	2.2 ± 0.2	1.2 ± 0.2	2.9 ± 0.4^§^	1.8 ± 0.3
$\dot{\text {V}}$O_2_, ml min^−1^ kg bw^−0.75^				
OVX	15.1 ± 0.3	13.6 ± 0.6	16.5 ± 0.9^§^	3.3 ± 1.3
OVX + E_2_	14.5 ± 0.6	12.1 ± 0.9	16.8 ± 0.4^§^	4.7 ± 0.7
D	13.3 ± 0.4	12.4 ± 0.7	14.1 ± 0.7^§^	1.9 ± 0.9
P	14.7 ± 0.3	14.0 ± 0.4	15.4 ± 0.7^§^	1.6 ± 0.9

Data are presented as means ± standard error

OVX, ovariectomized; E_2_, estradiol; D, control rats in the diestrus phase; P, control rats in the proestrus phase; *T*_a_, ambient temperature; *T*_abd_, abdominal temperature; *T*_tail_, tail skin temperature; bw, body weight; au, arbitrary unit

* Significant difference from the value in the OVX group, *P* < 0.05

^†^Significant difference from the value in the D group, *P* < 0.05

^§^ Significant difference between the light and dark phases, *P* < 0.05

### *T*_abd_ at *T*_a_ of 28 °C, 31 °C, and 34 °C in *Experiments 1 and 2*

**Fig. 2:**
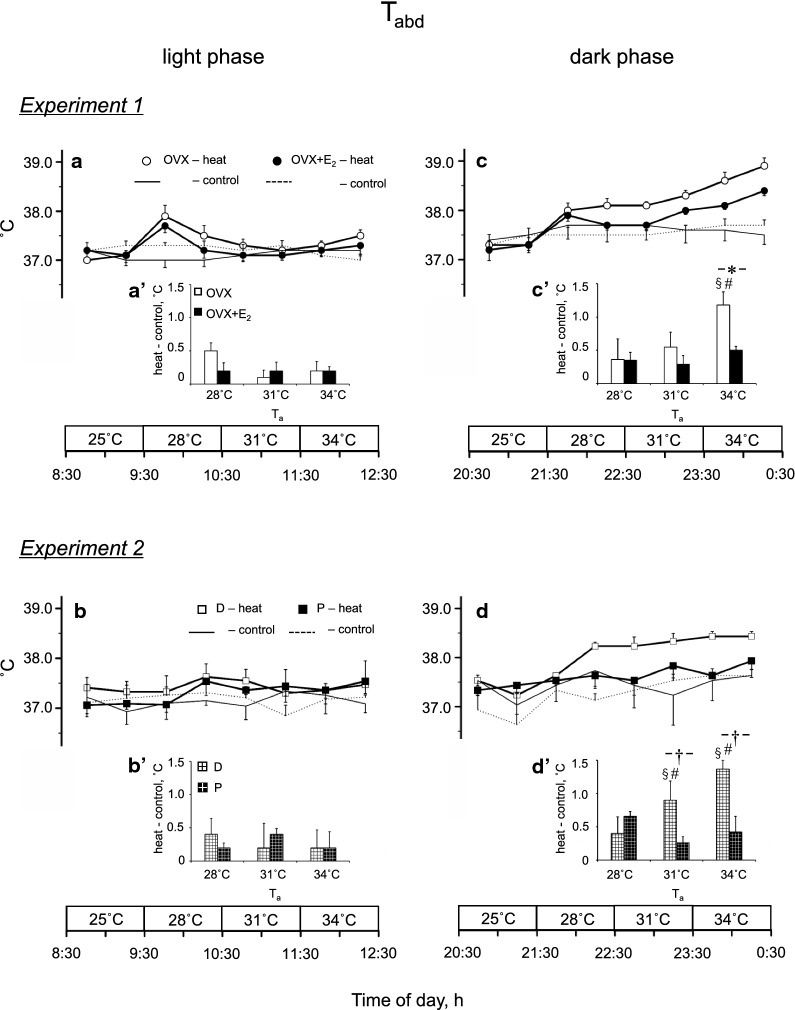
Abdominal temperature (*T*_abd_) during heat exposure in the ovariectomized (OVX) and OVX + estradiol (E_2_) groups (open and closed circles, respectively) in the light (**a**) and dark (**c**) phases, and in the D and P groups (open and closed squares, respectively) in the light (**b**) and dark (**d**) phases. The control data are indicated by solid and dashed lines without symbols (OVX and OVX + E_2_ groups in **a** and **c**, respectively, and D and P groups in **b** and **d**, respectively). Differences in *T*_abd_ at the same time of day between the heat exposure and control days are presented as H-C (**a**′–**d**′ in the light and dark phases in *Experiments* *1* and *2*, respectively). Data are presented as means ± standard error (**a** and **c**, *n* = 7 in each group; **b** and **d**, *n* = 5 in each group). *Significant difference between the OVX and OVX + E_2_ groups (*P* < 0.05). ^†^Significant difference between the D and P groups (*P* < 0.05). ^§^Significant difference between the light and dark phases (*P* < 0.05). ^#^Significant difference from the value at an ambient temperature (*T*_a_) of 28 °C (*P* < 0.05). D, control rats in the diestrus phase; P, control rats in the proestrus phase

Figure 2 shows *T*_abd_ in *Experiments* *1* and *2*. The difference in *T*_abd_ between the same period on the exposure and control days (*T*_abd_, H-C) is summarized as the 1 h average of each ambient condition (Fig. 2aʹ–dʹ). In *Experiment* *1*, there were significant effects of time [*P* = 0.003, *F*_(2, 44)_ = 6.77] and group [*P* = 0.012, *F*_(3, 22)_ = 4.63] with a significant interaction between the two factors [*P* = 0.002, *F*_(6, 44)_ = 4.23]. In the light phase, there were no differences in *T*_abd_, H-C between the OVX and OVX + E_2_ groups (Fig. 2aʹ). In the dark phase, *T*_abd_, H-C was greater in the OVX group than the OVX + E_2_ group at 34 °C (1.2 ± 0.2 and 0.5 ± 0.2 °C, *P* = 0.001; Fig. 2c′). In the OVX group, *T*_abd_, H-C was greater in the dark phase than the light phase at 34 °C (*P* < 0.001). In addition, in the dark phase, *T*_abd_, H-C at 34 °C was greater than that at 28 °C in the OVX group (*P* = 0.001).

In *Experiment* *2*, there were significant effects of time [*P* = 0.014, *F*_(2, 32)_ = 4.88] and group [*P* = 0.001, *F*_(3, 16)_ = 10.3] with a significant interaction between these two factors [*P* < 0.001, *F*_(6, 32)_ = 11.79]. In the light phase, there were no differences in *T*_abd_, H-C between the D and P groups (Fig. 2bʹ). In the dark phase, *T*_abd_, H-C was greater in the D group than the P group at 31 °C (0.9 ± 0.3 and 0.3 ± 0.0 °C, respectively, *P* = 0.018; Fig. 2dʹ) and 34 °C (1.4 ± 0.1 and 0.4 ± 0.2 °C, respectively, *P* < 0.001). In the D group, *T*_abd_, H-C was greater in the dark phase than the light phase at 31 °C and 34 °C (*P* = 0.02 and *P* < 0.001, respectively). In addition, in the dark phase, the *T*_abd_, H-C at 31 °C and 34 °C were greater than that at 28 °C in the D group (*P* = 0.001 and *P* < 0.001, respectively).

### *T*_tail_ at *T*_a_ of 28 °C, 31 °C, and 34 °C in *Experiments 1 and 2*

**Fig. 3:**
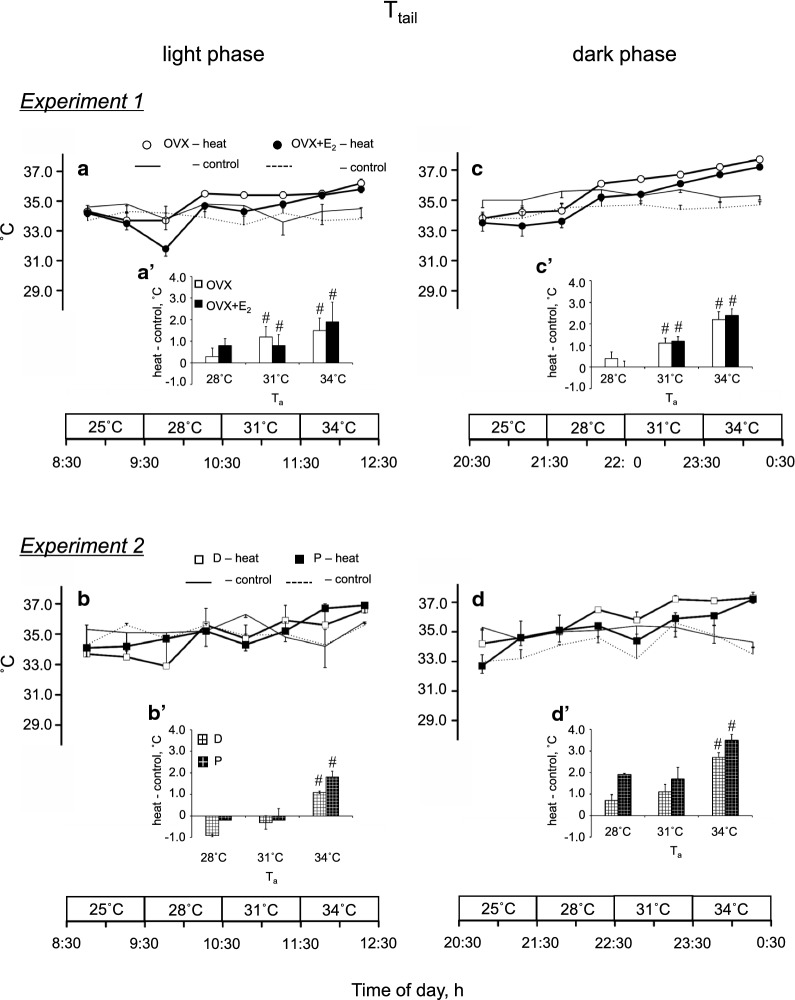
Tail skin temperature (*T*_tail_) during heat exposure in the ovariectomized (OVX) and OVX + estradiol (E_2_) groups (open and closed circles, respectively) in the light (**a**) and dark (**c**) phases, and in the D and P groups (open and closed squares, respectively) in the light (**b**) and dark (**d**) phases. The control data are indicated by solid and dashed lines without symbols (OVX and OVX + E_2_ groups in **a** and **c**, respectively, and D and P groups in **b** and **d**, respectively). Differences in *T*_tail_ at the same time of day between the heat exposure and control days are presented as H-C (**a**′–**d**′ in the light and dark phases in *Experiments* *1* and *2*, respectively). Data are presented as means ± standard error (**a** and **c**, *n* = 7 in each group; **b** and **d**, *n* = 5 in each group). ^#^Significant difference from the value at an ambient temperature (*T*_a_) of 28 °C (*P* < 0.05). D, control rats in the diestrus phase; P, control rats in the proestrus phase

Figure 3 illustrates *T*_tail_ in *Experiments* *1* and *2*. The difference in *T*_tail_ between the same period on the exposure and control day (*T*_tail_, H-C) is summarized in the same manner as *T*_abd_ (Fig. 3aʹ–dʹ). In *Experiment* *1,* there was a significant effect of time [*P* < 0.001, *F*_(2, 48)_ = 15.04]. In both phases, the *T*_tail_, H-C at 31 °C and 34 °C was greater than that at 28 °C in the OVX and OVX + E_2_ groups (*P* < 0.001, Fig. 3aʹ and c′). In *Experiment* *2*, there was a significant effect of time [*P* < 0.001, *F*_(2, 32)_ = 96.93]. In the two phases, the *T*_tail_, H-C at 34 °C was higher than that at 28 °C in the D and P groups (*P* < 0.001, Fig. 3bʹ and d′).

### $\dot{\text {V}}$O_2_ at *T*_a_ of 28 °C, 31 °C, and 34 °C in *Experiments 1 and 2*

**Fig. 4:**
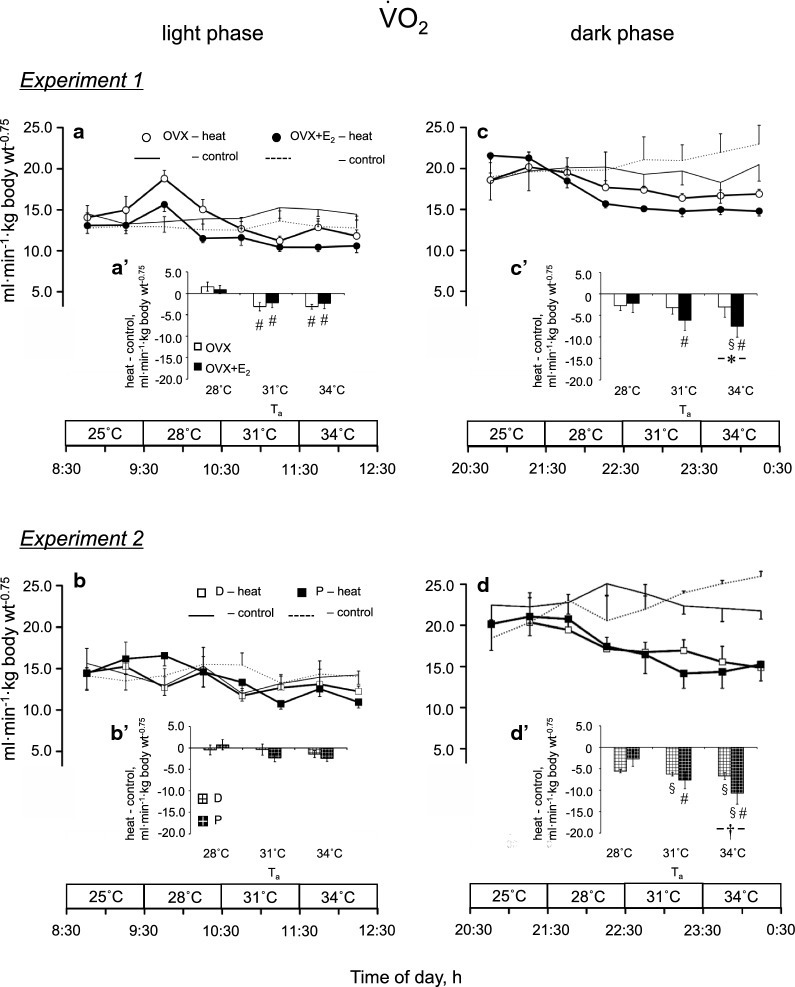
Oxygen consumption ($\dot{\text {V}}$O_2_) during heat exposure in the ovariectomized (OVX) and OVX + estradiol (E_2_) groups (open and closed circles, respectively) in the light (**a**) and dark (**c**) phases, and in the D and P groups (open and closed squares, respectively) in the light (**b**) and dark (**d**) phases. The control data are indicated by solid and dashed lines without symbols (OVX and OVX + E_2_ groups in **a** and **c**, respectively, and D and P groups in **b** and **d**, respectively). Differences in $\dot{\text {V}}$O_2_ at the same time of day between the heat exposure and control days are presented as H-C (**a**′–**d**′ in the light and dark phases in *Experiments* *1* and *2*, respectively). Data are presented as means ± standard error (**a** and **c**, n = 7 in each group; **b** and **d**, *n* = 5 in each group). *Significant difference between the OVX and OVX + E_2_ groups (*P* < 0.05). ^†^Significant difference between the D and P groups (*P* < 0.05). ^§^Significant difference between the light and dark phases (*P* < 0.05). ^#^Significant difference from the value at an ambient temperature (*T*_a_) of 28 °C (*P* < 0.05). D, control rats in the diestrus phase; P, control rats in the proestrus phase

Figure 4 indicates $\dot{\text {V}}$O_2_ in *Experiments* *1* and *2*. The difference in $\dot{\text {V}}$O_2_ between the same period on the exposure and control days ($\dot{\text {V}}$O_2_, H-C) is summarized in the same manner as *T*_abd_ (Fig. 4aʹ–dʹ). In *Experiment* *1*, there were significant effects of time [*P* < 0.001, *F*_(2, 48)_ = 21.40] and group [*P* = 0.038, *F*_(3, 24)_ = 3.28] with a significant interaction between these two factors [*P* = 0.006, *F*_(6, 48)_ = 3.49]. In the light phase, the $\dot{\text {V}}$O_2_, H-C at 31 °C and 34 °C was smaller than that at 28 °C in the OVX and OVX + E_2_ groups (*P* < 0.05, Fig. 4aʹ). In the dark phase, the $\dot{\text {V}}$O_2_, H-C was smaller in the OVX + E_2_ group than the OVX group at 34 °C (− 3.1 ± 2.3 and − 7.6 ± 2.5 ml min^−1^ kg body weight^−0.75^, *P* = 0.023, Fig. 4c′). In the OVX + E_2_ group, the $\dot{\text {V}}$O_2_, H-C was smaller in the dark phase than the light phase at 34 °C (*P* = 0.014). In addition, in the dark phase, the $\dot{\text {V}}$O_2_, H-C at 31 °C and 34 °C was smaller than that at 28 °C in the OVX + E_2_ group (*P* = 0.022 and *P* < 0.001, respectively).

In *Experiment* *2*, there were significant effects of time [*P* < 0.001, *F*_(2, 32)_ = 10.02] and group [*P* < 0.001, *F*_(3, 16)_ = 17.08]. In addition, a significant interaction between these two factors was observed [*P* = 0.034, *F*_(6, 32)_ = 2.63]. In the light phase, there were no differences in the $\dot{\text {V}}$O_2_, H-C between the D and P groups (Fig. 4bʹ). In the dark phase, the $\dot{\text {V}}$O_2_, H-C was smaller in the P group than the D group at 34 °C (− 6.7 ± 0.8 and − 10.7 ± 2.5 ml min^−1^ kg body weight^−0.75^, *P* = 0.019, Fig. 4d′). In the D group, the $\dot{\text {V}}$O_2_, H-C was smaller in the dark phase than the light phase at a *T*_a_ of 31 °C and 34 °C (*P* = 0.017 and *P* = 0.003, respectively). In the P group, the $\dot{\text {V}}$O_2_, H-C was smaller in the dark phase than the light phase at 34 °C (*P* < 0.001). In addition, in the dark phase, the $\dot{\text {V}}$O_2_, H-C was smaller at 31 °C and 34 °C than a *T*_a_ of 28 °C in the P group (*P* = 0.028 and *P* < 0.001, respectively).

### Spontaneous activity during the exposure at 28–34 °C in *Experiments 1* and *2*

Spontaneous activity did not change from that on the control day. There were no differences among the four groups in each phase.

### Heat loss index of the tail

**Fig. 5:**
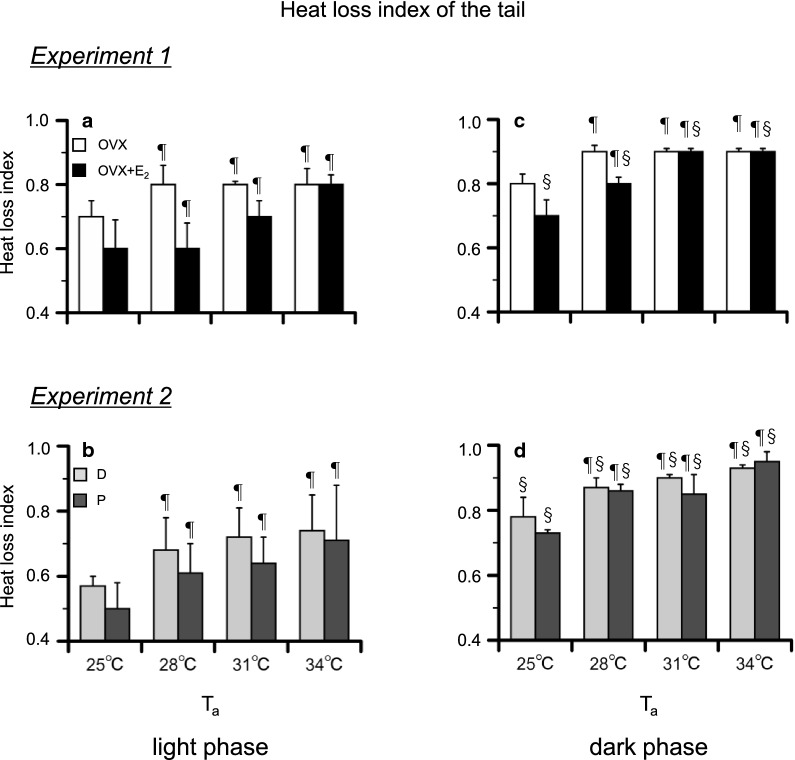
The heat loss index of the tail during heat exposure in the ovariectomized (OVX) and OVX + estradiol (E_2_) groups in the light (**a**) and dark (**c**) phases, and in the D and P groups in the light (**b**) and dark (**d**) phases. The heat loss index is presented as the 1 h average for each ambient temperature (*T*_a_). Data are presented as means ± standard error (*n* = 5 in each group). ^§^Significant difference between the light and dark phases (*P* < 0.05). ^¶^Significant difference from the value at a *T*_a_ of 25 °C (*P* < 0.05). D, control rats in the diestrus phase; P, control rats in the proestrus phase

The heat loss index of the tail at each *T*_a_ in *Experiments 1* and *2* is summarized in Fig. 5a–d. In *Experiment* *1*, there were significant effects of time [*P* < 0.001, *F*_(3, 72)_ = 37.56] and group [*P* < 0.001, *F*_(3, 24)_ = 19.50]. In both phases, the heat loss index at 28–34 °C was higher than that at 25 °C in both groups (*P* < 0.05). There were no significant differences between the two groups. In the OVX + E_2_ group, the heat loss index at 28–34 °C was higher in the dark phase than the light phase (*P* < 0.001).

In *Experiment* *2*, there were significant effects of time [*P* < 0.001, *F*_(3, 48)_ = 24.51] and group [*P* < 0.001, *F*_(3, 16)_ = 20.90]. In the two phases, the heat loss index at 28–34 °C was higher than that at 25 °C (*P* < 0.01) in both groups. No significant differences were found between the two groups. In both the D and P groups, the heat loss index was higher in the dark phase than the light phase (*P* < 0.001).

### Thermal conductance of the whole body

**Fig. 6:**
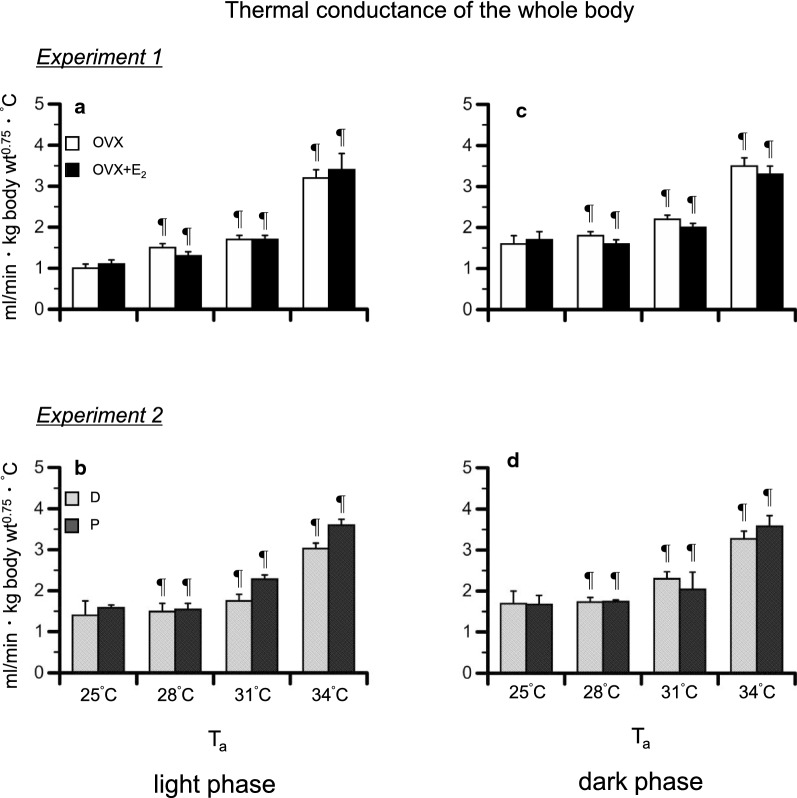
The thermal conductance of the whole body during heat exposure in the ovariectomized (OVX) and OVX + estradiol (E_2_) groups in the light (**a**) and dark (**c**) phases, and in the D and P groups in the light (**b**) and dark (**d**) phases. The thermal conductance is presented as the 1 h average for each ambient temperature (*T*_a_). Data are presented as means ± standard error (*n* = 5 in each group). ^¶^Significant difference from the value at a *T*_a_ of 25 °C (*P* < 0.05). D, control rats in the diestrus phase; P, control rats in the proestrus phase

The thermal conductance of the whole body in *Experiments* *1* and *2* is illustrated in Fig. 6a–d. In *Experiment* *1*, there were significant effects of time [*P* < 0.001, *F*_(3, 72)_ = 45.94] and group [*P* = 0.008, *F*_(3, 24)_ = 5.00]. In the light and dark phases, the thermal conductance at 28–34 °C was higher than that at 25 °C in both groups (*P* < 0.001, Fig. 6a, c). No significant differences were observed between the two groups in each phase.

In *Experiment* *2*, there was a significant effect of time [*P* < 0.001, *F*_(3, 48)_ = 30.42]. In both phases, the thermal conductance at 28–34 °C was higher than that at 25 °C (*P* < 0.01, Fig. 6b, d) in both groups. There were no significant differences between the two groups in each phase.

## Discussion

In the present study, we found that a higher level of plasma E_2_, which was observed in the OVX + E_2_ and P groups, which may activate thermoregulatory responses during exposure to 34 °C heat and prevent increases in *T*_abd_. However, such influence of E_2_ was observed only in the dark phase. In addition, attenuated metabolism in response to heat may be involved in the mechanism underlying the influence of E_2_.

### Body weight

It has been reported that ovariectomy augments increase in body weight, which is suppressed by E_2_ replacement [21–25]. In the present study, there were no differences in the final body weight in both phases between the OVX and OVX + E_2_ groups (Table 1). One possible reason is the variation of the initial body weight due to difference in the recovery period from the surgery (Table 1). Another reason may be that the duration of the E_2_ treatment may not have been long enough to influence body weight. It has been reported that the influence becomes apparent more than 4–5 weeks after the initiation of the E_2_ treatment [21, 22].

### *T*_abd_, *T*_tail_, spontaneous activity, and $\dot{\text {V}}$O_2_ on the control day

In both *Experiments* *1* and *2*, rats with higher level of plasma E_2_ (i.e., the OVX + E_2_ and P groups) exhibited a lower *T*_tail_ than the other groups only in the dark phase. However, this difference was not observed in *T*_abd_, spontaneous activity, or $\dot{\text {V}}$O_2_ (Table 2). *T*_abd_ became higher in the middle of the dark phase in the OVX + E_2_ rats, which may have reflected lower *T*_tail_ (i.e., attenuated heat loss). Although no statistical difference in the amplitude of $\dot{\text {V}}$O_2_, E_2_ may also increase $\dot{\text {V}}$O_2_ in the dark phase, resulting in grater *T*_abd_. As previously reported [11], E_2_ increased *T*_abd_ in OVX rats in the dark phase and *T*_tail_ inversely decreased (Fig. 1a, c). The result suggests difference in thermoregulatory control between the OVX and OVX + E_2_ groups even in the control condition. However, we did not find such difference between the P and D groups. This may be due to higher progesterone level in the P and D groups as previously reported [26]. Stachenfeld et al. [27] reported that the effect of E_2_ on thermoregulation is reversed by the presence of progesterone.

Previous studies have also reported an involvement of E_2_ in *T*_tail_, reflecting tail blood flow [11, 20, 28, 29]. However, the difference between the light and dark phases has not been well examined. In the present study, the OVX + E_2_ and P groups exhibited a higher level of plasma E_2_ than the other groups without any difference between the light and dark phases (Table 1). Only one study has reported a phase difference in the P phase, finding that the E_2_ level was lower in the early dark phase than the light phase [30]. Thus, at a *T*_a_ of 25 °C, the influence of E_2_ on *T*_tail_ may be modulated by the circadian phases.

Nagashima et al. [31] reported that the skin temperature of each part of the tail, as assessed by infrared thermography, reflects the heat loss response. This suggests that the *T*_tail_ data were reliable in evaluating heat loss in the present study. More importantly, the procedure does not disturb tail movement [11], which largely affects heat loss from the tail [32].

### Responses to the exposure at *T*_a_ of 28 °C, 31 °C, and 34 °C in the light phase

When rats are exposed to heat, heat loss processes are activated, such as vasodilation of the tail [33], saliva-spreading, grooming [6, 34, 35], posture change [36], and metabolism reduction [7, 8]. In this study, the heat loss index of the tail similarly increased from that at a *T*_a_ of 25 °C in each group in *Experiments* *1* and *2* (Fig. 5a, b). In addition, the thermal conductance of the whole body also similarly increased from that at a *T*_a_ of 25 °C in all groups (Fig. 6a, b). It was reported that, in rats, evaporative heat loss mechanism was activated when *T*_a_ surpasses of 34 °C [6]. Therefore, saliva-spreading and grooming may not be involved in the increase in the thermal conductance for the whole body. Moreover, E_2_ had no influence on the responses.

In *Experiment* *1*, a similar reduction of $\dot{\text {V}}$O_2_ from the control level was observed at a *T*_a_ of 31 °C and 34 °C in the OVX and OVX + E_2_ groups (Fig. 4a). It was reported that, in rats, the spontaneous activity decreased in 42 °C heat [9]. However, spontaneous activity did not change in the present study, suggesting that activity was not involved in the mechanism.

$\dot{\text {V}}$O_2_ did not change in the P and D groups in *Experiment* *2*. The difference from the result in *Experiment* *1* might be due to the higher progesterone level in the P and D groups. Uchida et al. [26] reported lower levels of plasma progesterone in OVX and OVX + E_2_ rats and a higher level in control rats in both the P and D phases. The difference would be because progesterone is secreted from the ovary. Nolan and Proietto [37] demonstrated that progesterone increased glucose uptake in the brown fat, which is associated with metabolic heat production. Thus, the higher progesterone level in the control rats may have maintained a greater $\dot{\text {V}}$O_2_ in the heat in a part.

### Responses to the exposure at *T*_a_ of 28 °C, 31 °C, and 34 °C in the dark phase

*T*_abd_ in the OVX and D groups was higher at 31 °C and 34 °C than at 28 °C; however, no changes were observed in the OVX + E_2_ and P groups (Fig. 2c, d). These results suggest the involvement of E_2_ in thermoregulation in response to heat. It was reported that E_2_ induces shift in thermoneutral zone to lower ambient temperature [2]. Thus, even the same *T*_a_ may have given greater thermal load to the OVX and D groups, increasing *T*_abd._

*T*_abd_ and *T*_tail_ at 20:30–0:30 (Fig. 1) was different from those on the control day in *Experiments* *1* and *2* (Figs. 2 and 3). One possible reason is that the data were assessed in the home cages, but the data on the control day were obtained in the Plexiglas box in the climatic chamber.

In *Experiment* *1*, heat loss responses, as assessed by the heat loss index of the tail and thermal conductance of the whole body, similarly increased in both groups (Figs. 5c and 6c). Therefore, heat loss responses do not explain for the increase of *T*_abd_ in the OVX and D groups.

$\dot{\text {V}}$O_2_ decreased from the control day only in the OVX + E_2_ group (Fig. 4c′). Because spontaneous activity was similar between the two groups, we concluded that activity was not involved in the mechanism. Several studies have reported that estrogen reduces energy intake [23, 38, 39]. Therefore, food deprivation during the heat exposure period may have caused the reduction in $\dot{\text {V}}$O_2_ via E_2_ in a part. However, this influence of E_2_ on energy intake may be small in the dark phase based on the findings of previous studies [24, 25]. Thus, we assume that the reduction in $\dot{\text {V}}$O_2_ was caused by the direct influence of the heat as part of the thermoregulatory responses. In addition, E_2_ may be involved in the mechanism. We did not assess ventilation in the present study, which may affect $\dot{\text {V}}$O_2_ and/or evaporative heat loss. Marques et al. [40, 41] reported that OVX rats showed lower ventilation only when either hypoxic or hypercapnia was applied. In addition, both E_2_ and P was not involved in the mechanism. Thus, we assume that E_2_ does not affect ventilation in heat. The mechanism underlying the difference in the reduction in $\dot{\text {V}}$O_2_ between the light and dark phases remains unclear, despite the similar levels of plasma E_2_ between phases. One possible reason is that $\dot{\text {V}}$O_2_ was sufficiently low in the light phase, which could not be a factor increasing *T*_abd_ in the heat.

We also found a similar increase in the heat loss index of the tail and the thermal conductance of the whole body in in the P and D groups in *Experiment* *2* (Figs. 5d and 6d). It was reported that progesterone has no influence on the tail skin temperature under ambient conditions [11]. Therefore, these results indicate that E_2_ and progesterone have no influence on the heat loss responses. In addition, we observed a difference in the reduction in $\dot{\text {V}}$O_2_ between the D and P groups in the dark phase (Fig. 4d′).

There are differences in the thermoregulatory responses between the OVX + E_2_ and P groups, suggesting the influence of progesterone. *T*_abd_ increased at *T*_a_ of 31 °C in the P group. At *T*_a_ of 31 °C, *T*_tail_ did not increase in both the P and D groups but increased in the OVX + E_2_ group. It was reported that progesterone per se increases body temperature; however, E_2_ reduces the effect [27]. Thus, even at *T*_a_ of 31 °C, the D group may not be able to control body temperature.

Previous study in rats reported a reduction of plasma thyroid stimulating hormones 7 days after 34 °C exposure [7]. Thus, thyroid function may be involved in the mechanism for the reduction of metabolism. However, we did not assess the thyroid function as well as the influence of E_2_. Thus, the mechanism remains unclear and needs to be clarified in future study.

## Conclusions

The present study suggests that, in both ovariectomized and control rats, a higher level of plasma E_2_ activates thermoregulatory responses to mild heat at 34 °C only in the dark phase. In addition, the decrease in metabolism in response to heat may be involved in the mechanism. The present study is the first to demonstrate the influence of E_2_ on metabolism as part of the thermoregulatory response to heat and the interaction with the circadian phase. However, the modulation of the metabolic response to heat by E_2_ remains unclear. This mechanism should be clarified in future studies. Moreover, our findings suggest that postmenopausal women experience reduced thermoregulation even in mild heat and are at higher risk of heat-related health problems.

## Data Availability

The datasets used and/or analyzed during the current study are available from the corresponding author on reasonable request.

## Abbreviations

ANOVA: Analysis of variance
D: Diestrus
E_2_: Estradiol
OVX: Ovariectomized
P: Proestrus
*T*_a_: Ambient temperature
*T*_abd_: Abdominal temperature
*T*_tail_: Tail skin temperature
$$\dot{\text {V}}$$O_2_: Oxygen consumption
